# Volatile biomarkers of symptomatic and asymptomatic malaria infection in humans

**DOI:** 10.1073/pnas.1801512115

**Published:** 2018-05-14

**Authors:** Consuelo M. De Moraes, Caroline Wanjiku, Nina M. Stanczyk, Hannier Pulido, James W. Sims, Heike S. Betz, Andrew F. Read, Baldwyn Torto, Mark C. Mescher

**Affiliations:** ^a^Department of Environmental Systems Science, ETH Zürich, 8092 Zürich, Switzerland;; ^b^Behavioural and Chemical Ecology Unit, International Centre of Insect Physiology and Ecology, Nairobi, Kenya;; ^c^Department of Biology, Pennsylvania State University, University Park, PA 16802;; ^d^Department of Entomology, Pennsylvania State University, University Park, PA 16802

**Keywords:** malaria, disease biomarkers, diagnostics, volatiles, asymptomatic infection

## Abstract

Malaria remains among the world’s deadliest diseases, and control efforts depend critically on the availability of effective diagnostic tools, particularly for the identification of asymptomatic infections, which play a key role in disease persistence and may account for most instances of transmission but often evade detection by current screening methods. Research on humans and in animal models has shown that infection by malaria parasites elicits changes in host odors that influence vector attraction, suggesting that such changes might yield robust biomarkers of infection status. Here we present findings based on extensive collections of skin volatiles from human populations with high rates of malaria infection in Kenya. We report broad and consistent effects of malaria infection on human volatile profiles, as well as significant divergence in the effects of symptomatic and asymptomatic infections. Furthermore, predictive models based on machine learning algorithms reliably determined infection status based on volatile biomarkers. Critically, our models identified asymptomatic infections with 100% sensitivity, even in the case of low-level infections not detectable by microscopy, far exceeding the performance of currently available rapid diagnostic tests in this regard. We also identified a set of individual compounds that emerged as consistently important predictors of infection status. These findings suggest that volatile biomarkers may have significant potential for the development of a robust, noninvasive screening method for detecting malaria infections under field conditions.

## Significance

Malaria elimination efforts are hindered by the prevalence of asymptomatic infections, which frequently go undetected and untreated. Consequently, there is a pressing need for improved diagnostic screening methods. Based on extensive collections of skin odors from human populations in Kenya, we report broad and consistent effects of malaria infection on human volatile emissions. Furthermore, we found that predictive models based on machine learning algorithms reliably determined infection status based on volatile biomarkers and, critically, identified asymptomatic infections with 100% sensitivity, even in the case of low-level infections not detectable by microscopy. These findings suggest that volatile biomarkers have significant potential for the development of robust, noninvasive screening methods for detecting symptomatic and asymptomatic malaria infections under field conditions.

In 2016, an estimated 216 million cases of malaria worldwide resulted in approximately 445,000 deaths (1). Sub-Saharan Africa was particularly hard hit, accounting for more than 90% of reported malaria cases and deaths, with most fatalities among children age <5 y (1). A key challenge for efforts to combat the spread of malaria is the fact that populations with high rates of exposure to *Plasmodium* parasites often harbor large numbers of individuals exhibiting partial immunity, who show few or no clinical symptoms despite being infected and capable of transmitting the parasite (2). Because asymptomatic cases typically go undetected and untreated (3), they constitute a hidden reservoir for the parasite that can contribute to the persistence of malaria transmission within localized populations, potentially accounting for up to 90% of onward transmission by vectors (4–6). Indeed, the prevalence of asymptomatic cases has recently been shown to have a positive correlation with transmission rates in regions exhibiting wide variation in overall disease prevalence, including Nigeria, Senegal, Gabon, and the Amazonian regions of Brazil (7). Therefore, identifying asymptomatic malaria cases is critical to efforts to effectively target drug treatment and other interventions to break the cycle of transmission (5, 8, 9).

Current diagnostic methods are poorly suited to large-scale screening of populations to identify individuals harboring asymptomatic infections, however (9). In particular, available screening techniques, such as microscopy and rapid diagnostic tests (RDTs), often fail to identify infections when parasite densities are low (4, 8), while more sensitive molecular-based methods entail the use of time-consuming and costly analyses and thus are not used as standard diagnostic tests. Furthermore, the recent discovery of *Plasmodium* spp. parasites with gene deletions that render them undetectable by widely used (HRP2-based) RDTs poses additional challenges for disease detection and raises broader questions about the potential evolution of diagnostic resistance (10, 11). Consequently, there is a pressing need for improved diagnostic methods capable of rapidly and reliably identifying asymptomatic infections under field conditions.

Volatile metabolites have been explored for diagnosis of a range of human diseases, including tuberculosis, cystic fibrosis, and cancer (12, 13), and there is reason to think that volatile biomarkers might prove particularly informative in the case of vector-borne pathogens, such as malaria, which may frequently manipulate host odors to attract vectors (14–16). In the case of malaria, previous work suggests that the parasite alters host odors in ways that influence mosquito behavior, and that such effects can occur in otherwise asymptomatic individuals. For example, previous work in humans has reported enhanced attraction of mosquito vectors to infected individuals that was likely mediated by as-yet-unidentified odor cues (17–19). Working in a mouse model, we documented similar mosquito attraction to infected but asymptomatic individuals and linked this behavior to characteristic changes in host odor profiles (20); moreover, another recent study also documented volatile changes associated with murine malaria (21). However, the effects of malaria on human volatiles remain largely undocumented, with small-scale clinical human trials yielding inconsistent results due to low participant numbers (22). Therefore, the present study aimed to characterize changes in human volatile emissions associated with symptomatic and asymptomatic malaria infections under field conditions to assess their potential value as diagnostic biomarkers.

## Results and Discussion

### Sample Collection and Determination of Malaria Infection Status.

**Fig. 1.**
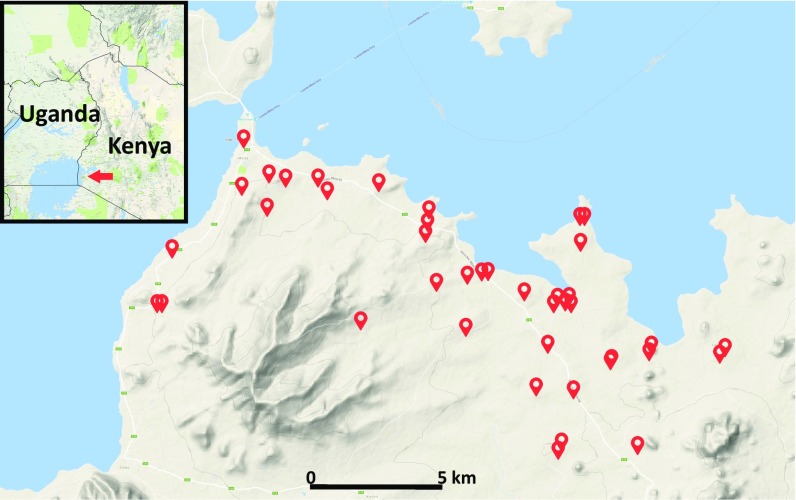
Volatile samples were collected from primary school children at 41 schools in western Kenya.

Between 2013 and 2016, we collected samples of skin volatiles from more than 400 primary-school children (aged ≤12 y) at 41 schools across 21 localities within the Mbita area of western Kenya (Fig. 1). Before sample collection, children were interviewed (using a standardized questionnaire) to assess medical history and current symptoms. Blood samples were then obtained from each participant, and skin volatiles were collected, separately but simultaneously, from one foot and one arm (at the elbow) for 1 h, using a portable push/pull volatile collection system. The blood samples were used for initial assessment of infection status via an SD Bioline Rapid Diagnostic Test (which detects malaria specific antibodies) and parasite detection by light microscopy. Children who tested positive were treated with artemether/lumefantrine. Because these diagnostic methods are imprecise—microscopy has a detection limit threshold, while RDTs have variable accuracy depending on malaria species and frequently yield false-positive results (11)—infection status was later definitively confirmed via PCR-based methods, which detect the presence of *Plasmodium* parasites with high sensitivity. (Additional details are provided in *Methods* and *SI Appendix*.) Based on these analyses and symptoms reported in the initial interview, subjects were classified as malaria uninfected (U), malaria symptomatic (S), or malaria infected but asymptomatic (AS). Subjects were included in our initial analyses only if both PCR and microscopy were negative (for U subjects) or positive (for S and AS subjects), yielding a total of 330 participants with unambiguous classifications. However, because this conservative approach excludes subjects harboring malaria infections who test negative via microscopy due to low parasite numbers—and because such individuals are important from a diagnostic perspective—we included 66 individuals who were positive by PCR but negative by microscopy in some subsequent analyses. We refer to these subjects as submicroscopic symptomatic (S_[SUB]_) or submicroscopic asymptomatic (AS_[SUB]_).

### Analysis of Volatile Profiles.

Volatile samples were analyzed by gas chromatography-mass spectrometry. Only compounds that exceeded a minimum concentration threshold in ≥75% of the samples for at least one category of infection status (U, S, or AS) were included in our subsequent analyses. The resulting data were explored using machine learning techniques (discussed below) to identify predictors of infection status. In subsequent discussion, the overall dataset is treated as two independent subsets, Kenya 1 (K1) and Kenya 2 (K2), due to differences in the chemical analyses used (owing to a change of institution by the lead investigator during the study and the resulting availability of more sensitive analytical equipment). K1 comprises 95 U, 99 S, and 34 AS individuals, and K2 comprises 39 U, 35 S, and 28 AS individuals. Volatile profiles for the additional 66 individuals classified as malaria-negative by microscopy but as malaria-positive by PCR (52 S_[SUB]_ and 14 AS_[SUB]_) were analyzed by the methods used for K2 but are presented separately below except where noted otherwise.

Because the chemical analytical techniques used for K2 allowed for much higher-quality analyses (due to an approximate 25-fold increase in sensitivity, as well as improved compound separation), our analyses focus primarily on this subset of the data; however, we discuss similarities and differences between the two datasets where appropriate. In general, there is broad agreement in the overall patterns revealed by the two datasets, even though they were derived from separate sets of samples that were analyzed on different equipment at different locations.

### Volatile Profiles and Infection Status.

**Fig. 2.**
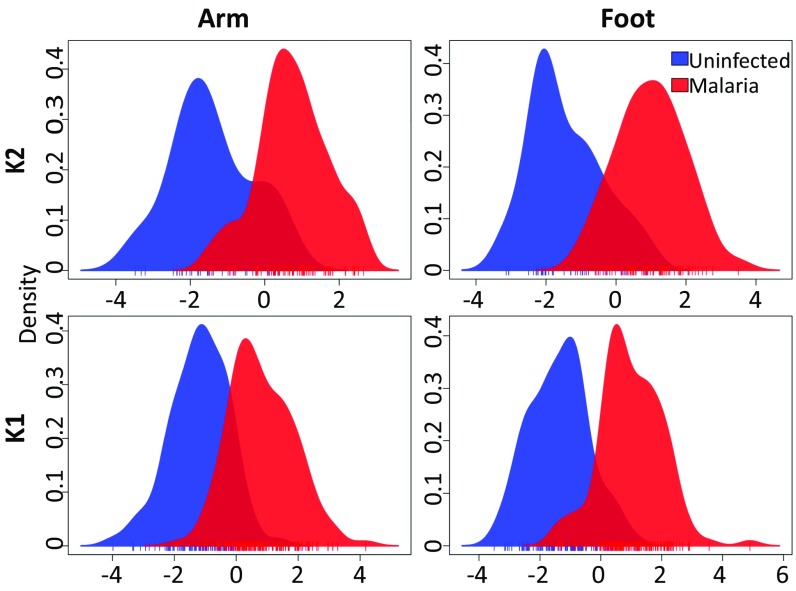
Group separation using DAPC reveals differences between malaria-infected (asymptomatic + symptomatic) and uninfected individuals in foot and arm volatiles for datasets K1 and K2. Vertical lines beneath the *x*-axis represent individual samples.

**Fig. 3.**
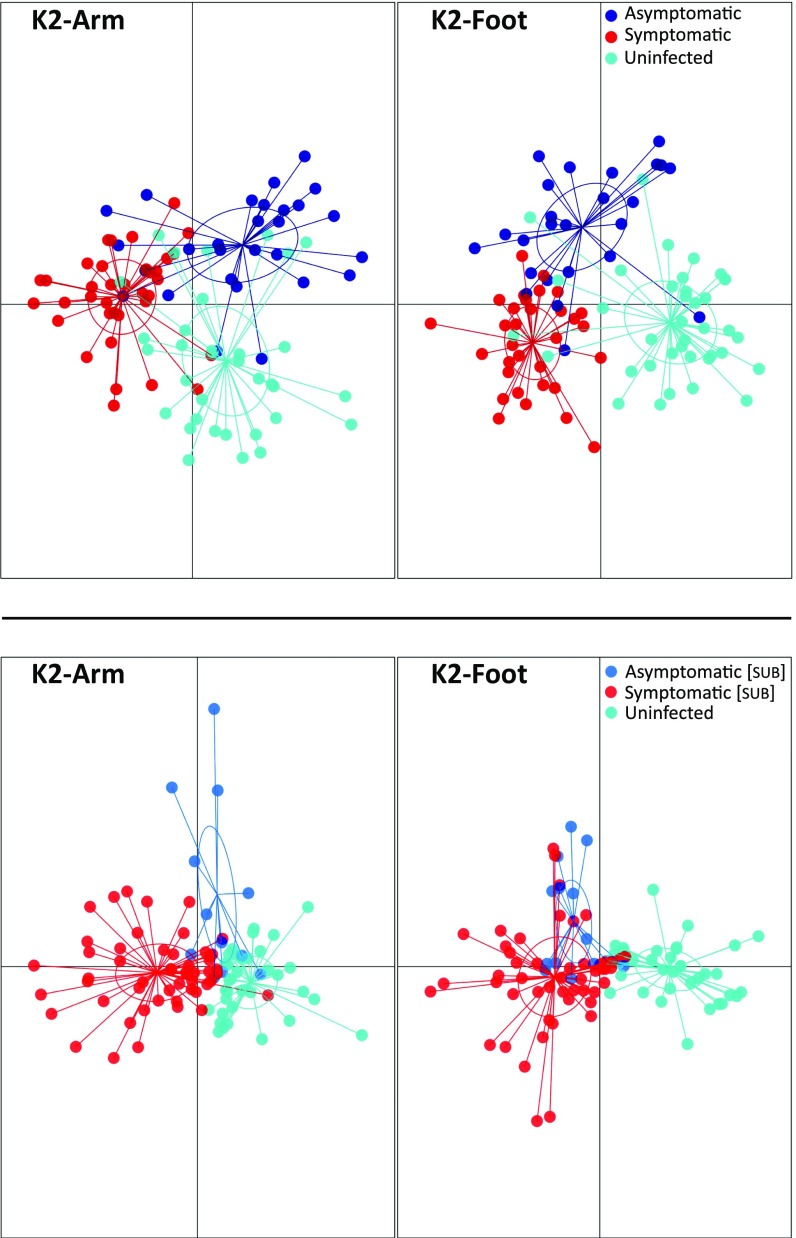
Group separation using DAPC for K2 arm and foot volatiles. (*Top*) Differences among uninfected individuals and individuals with symptomatic and asymptomatic malaria infections, confirmed by both microscopy and PCR. (*Bottom*) Differences among uninfected individuals and individuals with submicroscopic symptomatic and asymptomatic infections, detected only by PCR. Points represent individual samples, with colors denoting malaria condition and inclusion of 95% inertia ellipses.

Initial investigation of the data via discriminant analysis of principal components (23) (DAPC) revealed separation between the volatile profiles of malaria-infected individuals (including both S and AS subjects) and uninfected individuals for both foot and arm (Fig. 2). This separation was apparent for both K1 and K2 but was more pronounced for K2, likely reflecting the improved quality of the chemical analyses. Incorporating symptom status in our analyses also revealed separation between the volatile profiles of S and AS individuals, as well as between each of these groups and U individuals (Fig. 3). Once again, this pattern was also apparent but less pronounced for K1 (*SI Appendix*, Fig. S1). We also observed similar separation of the volatile profiles of S_[SUB]_ and AS_[SUB]_ individuals from U individuals (Fig. 3), suggesting that even submicroscopic malaria infections generate a volatile signature.

**Fig. 4.**
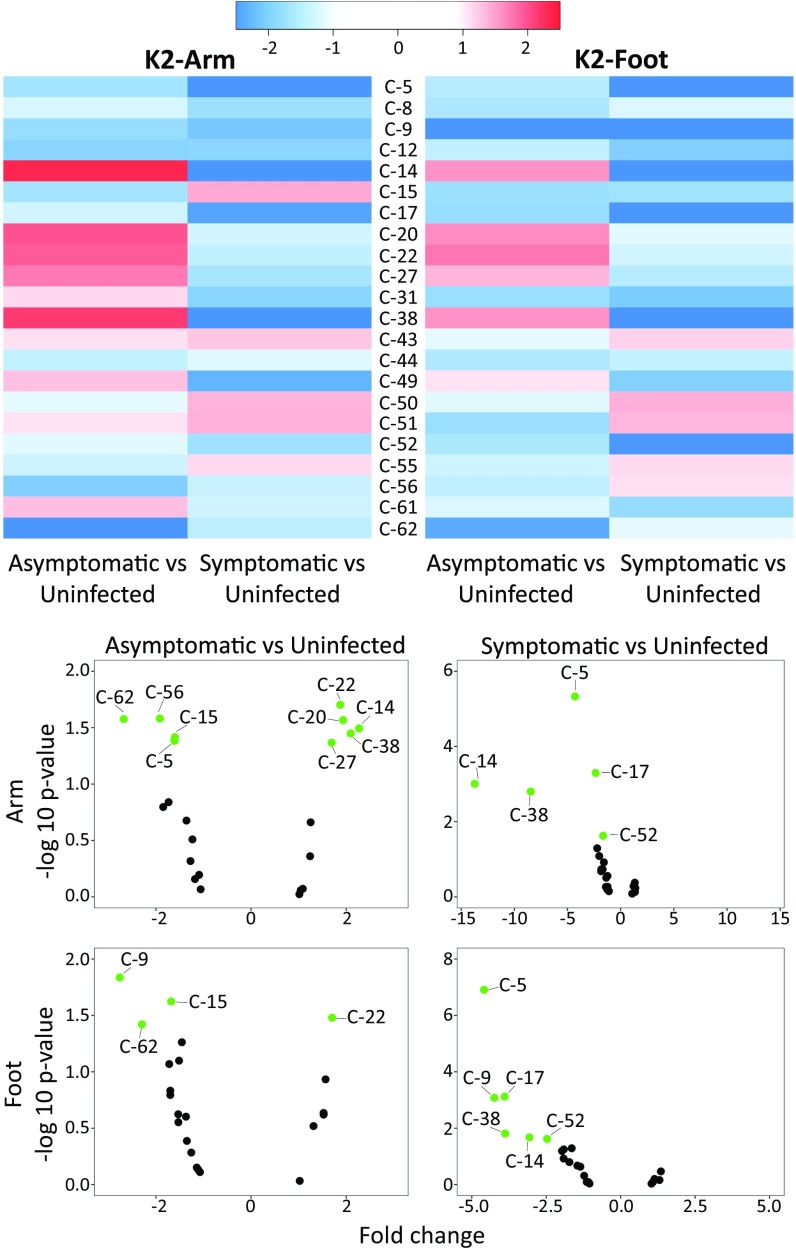
(*Top*) Heatmap showing the fold-change of individual compounds in asymptomatic and symptomatic individuals relative to those in uninfected individuals for K2-arm and K2-foot. (*Bottom*) Volcano plots showing changes in individual compounds in asymptomatic and symptomatic individuals, relative to those in uninfected individuals, with compounds significantly up- or down-regulated shown in green (*P* < 0.05 and absolute fold change >1.5). Nonsignificant regulated compounds with absolute fold change <1.5 shown in black. (Compound IDs are listed in Table 2.)

The divergent effects of S and AS infections on volatile emissions were also apparent when comparing changes in the levels of individual compounds relative to those observed for U individuals (Fig. 4 and *SI Appendix*, Fig. S2). In general, the effects of infection status on individual compounds tended to be similar in arm and foot samples (Pearson’s *r* = 0.8 for asymptomatic vs. uninfected and 0.61 for symptomatic vs. uninfected). Emission levels of most compounds were reduced in S individuals, with several compounds showing strong suppression. Effects on the emissions of AS individuals were more mixed, with some compounds elevated and others suppressed relative to the levels observed for U individuals. In general, the strongest suppression was observed for compounds in S individuals and the strongest up-regulation was seen for compounds in AS individuals. This overall pattern is consistent with our previous observation that volatile emissions were suppressed during the acute phase of infection by the rodent malaria parasite *Plasmodium chabaudii* in a mouse model (20).

### Predictive Models of Infection Status.

To characterize the volatile signatures associated with each category of infection status, we used machine learning algorithms that develop tree-based ensemble classification models, which aim to identify a minimal set of compounds that correctly classify individuals. These algorithms were used to “train” models on 70% of samples from K2, iteratively eliminating the least important compounds (i.e., those making the smallest contribution to accuracy) to obtain a subset resulting in the best model performance, which was then tested on the remaining 30% of samples. We found that adaptive boosting (adaboost) consistently provided the best predictions for our data with respect to the key metrics of sensitivity (i.e., proportion of malaria-positive individuals detected) and accuracy (i.e., proportion of all cases classified correctly). Given our focus on identifying diagnostically meaningful biomarkers of infection, our model selection emphasized sensitivity over accuracy (i.e., we are willing to accept a somewhat higher rate of false-positives to maximize the proportion of actual malaria cases correctly identified), and also favored models capable of generating predictions using relatively few compounds. As noted, we are also particularly interested in the ability to identify asymptomatic (AS and AS_[SUB]_) infections.

Despite the overall similar effects of infection status on arm and foot volatiles, predictive models using foot volatiles exhibited greater sensitivity, particularly for the detection of asymptomatic infections. Model sensitivity and accuracy for several key comparisons, along with the compounds used as predictors, are presented in Table 1. Models based on foot volatiles identified AS infections with 100% sensitivity and S infections with 91% sensitivity. Furthermore, these models also identified submicroscopic S_[SUB]_ and AS_[SUB]_ infections with 100% sensitivity. This performance greatly exceeded that of our RDT screening, which failed to identify more than one-third of AS_[SUB]_ infections and approximately one-fourth of S_[SUB]_ infections (*SI Appendix*, Table S1). Although we observed differences in the effects of symptomatic and asymptomatic infections on volatile profiles, we also tested the ability of our algorithms to detect all malaria cases without regard to symptom status and including submicroscopic infections. Here our model was able to predict infection status with 95% sensitivity and 77% accuracy using foot volatile compounds, or with 92% sensitivity and 80% accuracy using arm volatiles.

**Table 1.** Key predictors of infection status

	S vs. U		AS vs. U		S_[sub]_ vs. U		AS_[sub]_ vs. U		Infected (all) vs. U	
	Foot	Arm	Foot	Arm	Foot	Arm	Foot	Arm	Foot	Arm
Sensitivity, %	91	89	100	75	100	80	100	100 (90)	95	92
Accuracy, %	85	89	78	78	100	88	100	100 (92)	77	80
Top predictors	C-49	C-56	C-43	C-49	C-5	C-5	C-5	C-5	C-17	C-56
	C-9	C-5	C-56	C-56	C-20	C-20	C-17	C-20	C-49	C-61
	C-5	C-22	C-61	C-31	C-17	C-15	C-20	C-52	C-31	C-5
	C-43	C-17	C-5	C-20	C-14	C-52	C-9	C-8	C-61	C-51
	C-17	C-52	C-49			C-14		C-56	C-5	C-31
			C-31					C-62		
			C-17					C-15		
			C-44					C-31		

For each comparison, compounds are listed in order of importance for the predictive model. Compound IDs are provided in Table 2. Numbers in parentheses show model sensitivity/accuracy when using only the top four predictors.

Overall, the results of our predictive models suggest the presence of volatile signatures that can reliably predict malaria infection status and, critically, identify AS malaria infections with high sensitivity, even in the case of incipient or otherwise low-level infections.

### Selected Key Compounds.

Chemical identities for all the compounds included in our analyses are presented in Table 2. To illuminate key compounds with potential diagnostic significance, we focused on those that were consistently found to be important predictors of infection status by our machine learning algorithm (adaboost) (24). As noted above, models based on foot volatiles generally exhibited the best predictive performance. For general prediction of malaria infection status (without regard to symptom status), the five most important foot volatiles for model accuracy were compound 17 (4-hydroxy-4-methylpentan-2-one), compound 49 (unidentified), compound 31 (unidentified), compound 61 (nonanal), and compound 5 (toluene) (Table 1). These five compounds also frequently appeared as important predictors across other comparisons. For example, each was an important predictor for models predicting both AS vs. U and S vs. U on the basis of either foot or arm volatiles (or both) (Table 1).

**Table 2.** Compound IDs and selected key compounds

Compound no.	Compound ID
**C-5**	**toluene**
C-8	octane
**C-9**	**hexanal**
C-12	2,4-dimethylheptane
**C-14**	**ethylcyclohexane**
C-15	2,4-dimethylhept-1-ene
**C-17**	**4-hydroxy-4-methylpentan-2-one**
**C-20**	**ethylbenzene**
C-22	m-xylene or p-xylene
C-27	*o*-xylene
**C-31**	**Unidentified**
**C-38**	**propylcyclohexane**
C-43	1-ethyl-3-methylbenzene
C-44	benzaldehyde
**C-49**	**Unidentified**
C-50	1,2,4-trimethylbenzene
C-51	decane
C-52	octanal
C-55	*s*(-)-limonene
**C-56**	**2-ethylhexan-1-ol**
**C-61**	**nonanal**
C-62	dodecane

Boldface text indicates key compounds that were consistently important predictors in our models and/or exhibited notable emission patterns (as discussed in the text).

Compound 56 (2-ethylhexan-1-ol) also frequently appeared as an important predictor for both asymptomatic and symptomatic infections and was the top predictor for overall infection status for arm volatiles (Table 1), while compound 20 (ethylbenzene) consistently appeared as an important predictor of submicroscopic infections. Compound 14 (ethylcyclohexane) appeared as a predictor in some models and, along with compound 38 (propylcyclohexane), exhibited an interesting pattern in which its emission exhibited relatively strong up-regulation in AS individuals and relatively strong suppression in S individuals (Fig. 4). Finally, compound 9 (hexanal), which also appeared as a predictor in some models, exhibited relatively strong suppression in both S and AS individuals, particularly in foot volatile profiles (Fig. 4).

Two of the compounds discussed above, toluene and hexanal, are notable in that they have previously been reported to be produced by *Plasmodium* parasites in vitro (25, 26). In addition, hexanal, ethylbenzene, 2-ethylhexan-1-ol, and nonanal have previously been shown to elicit electrophysiological responses in mosquitoes (27–29) (*SI Appendix*, Table S3). We previously suggested that parasite-induced changes in host odors that have relevance for vector behavior might provide reliable biomarkers for disease diagnosis (20), raising the possibility of overlap between compounds that are predictive of infection status and those that influence vector behavior. A search of the existing literature suggests that mosquito responses have been examined for relatively few of the compounds mentioned above (*SI Appendix*, Table S3); however, we plan to explore the influence of relevant changes in the emissions of these and other compounds on vector behavior in a future study.

### Origins and Emission Patterns of Key Compounds.

Although some of the compounds discussed above can occur as environmental contaminants (e.g., toluene, 4-hydroxy-4-methylpentan-2-one, and 2-ethylhexan-1-ol) (30, 31), all of the identified compounds highlighted here have either been previously reported from human volatile collections or have known mechanisms of natural production from humans or potentially human-associated microbes (*SI Appendix*, Table S4). Compounds originating from human-associated microbiota are of significant diagnostic interest, particularly with respect to the identification of volatile biomarkers, and previous work in a mouse model has documented the effects of malaria infection on the microbiome (32, 33). Furthermore, one of the most consistently important predictors in our models, compound 5 (toluene)—which may be produced by *Clostridium* in the human microbiome (34)—has previously been explored as a diagnostic biomarker for cancer in humans (35).

To confirm that the key compounds discussed above (and highlighted in Table 2) are directly affected by infection status and exclude potential biases (e.g., due to spatiotemporal coincidence of environmental contamination and high rates of infection), we further explored how emission levels of these compounds varied between uninfected and infected individuals across individual sample collection sessions. Linear analysis via two-way ANOVA revealed significant variation in compound levels across collection sessions (*SI Appendix*, Table S5), as expected given that volatile emissions are labile and highly responsive to environmental conditions (36). However, these analyses also revealed a highly significant effect of infection status (S + AS vs. U) for all of our key compounds except compound 61 (nonanal) (*SI Appendix*, Table S5). There was also a significant interaction effect between collection session and infection status (*SI Appendix*, Table S5), which might be explained if, for example, infection influences the responsiveness of emissions to other environmental influences; however, this interaction effect does not obscure the effects of disease status. These analyses confirm that malaria infection affects the emissions of our key predictors even when accounting for environmental variation across collection sessions.

## Conclusions

Our results show that malaria infection causes broad and consistent changes in human volatile emissions. Furthermore, we found consistent differences in the effects of symptomatic and asymptomatic infection on human volatile profiles. These changes in volatile profiles create a signature of infection that can be used to reliably predict the infection status of human subjects. Critically, these volatile signatures can identify asymptomatic infections with high sensitivity, even in the case of low-level infections not detectable by microscopy—a key finding given the pressing need for more effective diagnostic methods capable of detecting asymptomatic carriers of infection (4, 5, 8, 9). Indeed, our predictive models performed significantly better than RDT, and as well as PCR, in detecting submicroscopic infections. It is also important to note that this performance was achieved based on the analysis of volatile samples collected under field conditions and despite considerable variation introduced through sampling that took place over 3 y and across a number of different localities, as well as by the presence of multiple *Plasmodium* species and a high prevalence of mixed parasite infections in our study population. Finally, our analyses highlight a number of key compounds that consistently appear as important predictors of infection across our predictive models and thus warrant further exploration as biomarkers with potential for the development of robust, noninvasive volatile-based diagnostics for malaria infection.

## Methods

### Sample Collection.

#### Participant selection.

Participant exclusion criteria included (*i*) receipt of antimalarial medication during the previous 2 wk; (*ii*) chronic disease, such as HIV; (*iii*) not signing (or having a parent sign) the consent form; and (*iv*) refusal of malaria treatment.

#### Ethical approval.

This study was approved by The Pennsylvania State University (IRB #41529), ETH Zürich (EK2015-*N*-59), and the Kenya Medical Research Institute (SERU 391). Before sample collection, the study and consent form were explained to parents/guardians and their written informed consent was obtained.

#### Malaria infection status.

Infection status was initially assessed by RDT (SD Bioline) and light microscopy. Three blood spots were collected on filter paper for later confirmation of infection status by nested PCR (37), which also provided information about *Plasmodium* species. The species present were *P. falciparum*, *P. ovale*, and *P. malariae*; the majority of children presented with mixed infections, most often including *P. falciparum*. Recent symptoms (and other aspects of medical history) were assessed in an initial interview using a standardized questionnaire. Symptoms indicative of malaria included fever, abdominal pain, rash, diarrhea, vomiting, and body aches. Children found to be positive for malaria by RDT were started on a 3-d regimen of artemether/lumefantrine after confirmation by light microscopy. Additional information is provided in *SI Appendix*.

#### Volatile collections.

Volatiles were collected (prior to treatment of infected individuals) simultaneously for 1 h from one arm (wrist to above the elbow) and one foot (to above the ankle), using a portable system (PVAS22; Volatile Assay Systems). Teflon sleeves for arms and bags for feet (American Durafilm), were closed with Velcro strips. Carbon-filtered air was pushed through an entry port (feet: 1.8 L/min; arms: 1.1 L/min) and pulled through an exit port (feet: 1.1 L/min; arms: 0.8 L/min), where volatiles were collected on HayeSep adsorbant polymer filters (80/100 mesh; Millipore Sigma). The volume of air collected was taken into account in subsequent analyses.

### Chemical Analyses.

#### Sample preparation.

Each sample was eluted by adding 150 µL of dichloromethane (HPLC grade) and flushing with a gentle stream of nitrogen gas. *p*-Bromofluorobenzene (99%; Millipore Sigma) was added as an internal standard at a final concentration of 6 ng/μL.

#### Compound quantification and identification by GC-MS.

Data subset K1 was analyzed on an Agilent 5973 mass spectrometer coupled to a 6890 gas chromatograph, a setup capable of identifying a signal-to-noise ratio of 60:1. K2 was analyzed on an Agilent MSD 5977A mass spectrometer coupled to a 7890B gas chromatograph, capable of identifying a signal-to-noise ratio of 1,500:1. The analyses for K2 were therefore approximately 25-fold more sensitive than those available for K1; furthermore, differences in instrument configuration (resulting in a lower flow rate for K2) led to much better separation of individual compounds in K2. For analysis of K2, compounds from a 2.5-µL injection were separated on an Agilent HP-5 ms capillary column (30 m × 0.25 mm i.d. × 0.1 μm film thickness), using the following temperature program: 35 °C for 0.5 min then raised at 7 °C/min to 270 °C and a constant flow rate of 0.9 mL/min of helium. Compounds were detected with an electron impact single quadrupole mass spectrometer (70 eV; ion source 230 °C; quadrupole 150 °C; mass scan range, 30–350 amu). This system allows simultaneous analysis with a flame ionization detector; however, flame ionization-based quantification was not possible due to multiple coeluting compounds. Details of the chemical analysis of K1 are provided in *SI Appendix*.

Chemical data were processed using the MassHunter software suite (Agilent). An initial list of 186 compounds was generated using an automated tool in MassHunter’s quantitative analysis package, with peak identification requirements of an absolute area of 500 counts, a signal intensity of 500 counts, and a signal-to-noise ratio of 2. A single sample was used to generate the initial list of compounds, and additional compounds encountered were added to the list. Each compound was assigned a characteristic ion; if multiple compounds were assigned the same ion and retention time (0.05-min window), the duplicate was removed. Initial identification of selected compounds was carried out using MassHunter’s qualitative analysis package and the NIST14 chemical library. Compounds of interest, as determined by statistical analysis, were further verified by comparison with external standards (Sigma Aldrich and TCI Deutschland) (*SI Appendix*, Table S2).

### Analyses of Volatile Profiles.

#### Datasets and compound exclusion.

As noted above, K1 and K2 were analyzed on different equipment at different locations. Furthermore, samples for K1 and K2 were collected during different periods and from only partially overlapping geographical locations. Consequently, K1 and K2 were treated separately in our analyses. Foot and arm volatiles were also analyzed separately. Compounds were excluded if not found above a set concentration (0.04 ng/μL relative to the internal standard) in at least 75% of the samples for at least one category of infection status (AS, S, or U). The K1 dataset comprised 95 U, 101 S, and 34 AS individuals (Fig. 2), and the K2 dataset comprised 39 U, 35 S, 29 AS, 53 S_[SUB]_, and 14 AS_[SUB]_ individuals (Figs. 2–4).

#### DAPC.

DAPC (23) was used to visualize differences among malaria status groups based on discriminant functions. This method maximizes differences between groups while minimizing variation within clusters. It uses principal component analysis to transform the data into uncorrelated variables before discriminant analysis. Additional details are provided in *SI Appendix*.

#### Heat maps and volcano plots.

Heat maps and volcano plots were constructed to visualize differences in the volatile emissions of malaria-infected individuals (S and AS) relative to those of uninfected individuals (U). These visualizations compare the mean of individual compounds in foot and arm samples from infected individuals to the corresponding mean for uninfected samples and display the results as fold change. In addition, compounds that differ significantly (*P* < 0.05) between asymptomatic or symptomatic relative to uninfected individuals are highlighted in the volcano plots. A Pearson correlation coefficient was computed between arm and foot samples to compare the consistency of changes across infection status.

#### Predictive models.

The variable selection process focused on selecting an optimal classification model with high sensitivity and accuracy, while identifying the minimal number of compounds needed for effective prediction. We partitioned K2 into a training set and a test set using 70% and 30% of the K2 data, respectively. Using the training set, we trained three machine learning classification algorithms—random forest (rf) (38), regularized random forest (rrf) (39), and adaptive boosting (adaboost) (24)—using a recursive feature elimination algorithm implemented with the *rfe* function in the R *caret* package (40) with inner resampling using a 10-fold cross-validation to tune the classification model at each iteration. These algorithms use all compounds included in the data analyses to fit an initial model on the training set, with each compound ranked according to its importance in successfully categorizing malaria status. The resulting model is iteratively reduced, removing the least important compound each round until a subset resulting in the best accuracy is determined. This subset of compounds is then used to generate the final model on the test set. Because we found that adaboost consistently produced the greatest accuracy and sensitivity in detecting malaria cases across all categories in the test set, this algorithm was used for all models presented in the paper. Parameters (adaboost: *mfinal* and *maxdepth*; rrf: *mtry*; rf: *mtry*) were tuned away from their default parameter settings, and the values that resulted in the most accurate models were used to train the final versions of the models reported. Each model produced a list of compounds required to obtain a given accuracy and sensitivity on the test set. To test whether models could predict infection status using fewer compounds, we used the top 5 or 10 predictors from each model as the basis for a new model, resulting in new accuracy and sensitivity for the simplified model (Table 1). Additional details are provided in *SI Appendix*.

#### Linear analysis.

Further linear analysis was used to obtain the statistical significance of the compounds selected in the predictive model step. We used the function lmFit from the R package limma (41) to run individual *t* tests on each compound. To refer to a result as “statistically significant,” we used a *P* value <0.05 and a fold-change of 1 higher or lower than the base of comparison. To further explore the date effect or selected compounds, we performed a two-way ANOVA in the R package *ARTool* (42), with infection status (AS + S vs. U) or (S vs. U) and collection date as the main effects. All collections on a given date took place at a single location.

All data analysis and further visualization were done in in R version 3.3.3 (43). Additional details of the statistical analyses are provided in *SI Appendix*.

## Supplementary Material

Supplementary File
