# Supplementary material for: Volatile biomarkers of symptomatic and asymptomatic malaria infection in humans
Source: Proc Natl Acad Sci U S A. 2018 May 14;115(22):5780–5. doi: 10.1073/pnas.1801512115 (PMC5984526; doi:10.1073/pnas.1801512115)
Supplement: Supplementary File [file pnas.1801512115.sapp.pdf]

SUPPORTING INFORMATION

Volatile biomarkers of symptomatic and asymptomatic malaria infection in humans

Authors: Consuelo M. De Moraes[1], Caroline Wanjiku[2], Nina M. Stanczyk[1], Hannier Pulido[1], James W. Sims[1], Heike S. Betz[3], Andrew F. Read[4], Baldwyn Torto[2], Mark C. Mescher[1†]

Affiliations:

1. Department of Environmental Systems Science, ETH Zürich, Zürich, 8092, Switzerland
2. Behavioural and Chemical Ecology Unit, International Centre of Insect Physiology and Ecology, P.O. Box 30772-00100, Nairobi, Kenya.
3. Department of Biology, Pennsylvania State University, University Park, PA 16802, USA.
4. Departments of Biology and Entomology, Pennsylvania State University, University Park, PA 16802, USA.

[†]To whom correspondence should be addressed:

Mark C. Mescher, Department of Environmental Systems Science, Institute of Integrative Biology, LFO G19, ETH Zürich, 8092 Zürich, Switzerland
Email address: mescher@usys.ethz.ch, Telephone number: +41 44 632 39 20, Fax: +41 44 632 11 71

Keywords: malaria, disease biomarkers, diagnostics, volatiles, asymptomatic infection

Supplemental methods:

Determination of malaria status

Light Microscopy: Thin and thick blood smears were prepared for microscopy and stained in 10% Giemsa prepared using acid buffered water (pH 7.2). The films were then read in duplicate under high power magnification in oil immersion on a light microscope (Optika B-192) by two independent, experienced microscopists. Parasite counts were enumerated per every 200 white blood cells (WBCs) on the thick film. Parasite species were identified using the thin film.

Rapid Diagnostic Tests (RDT): Tests employed the SD Bioline™ kit (Standard Diagnostic, INC.) in accordance to manufacturer's instructions. The kit provides identification of *Plasmodium falciparum* malaria based on a specific antibody reaction (histidine-rich protein II), and also a non-specific enzyme-based test (*Plasmodium* lactate dehydrogenase) for the presence of any other malaria species present in the area (*P. ovale* and *P. malariae*).

DNA extractions: Three blood spots were collected on qualitative filter paper for confirmation of infection status and infecting species by nested PCR coupled to high resolution melting (HRM) targeting the 18S rRNA gene in a method adapted from Kipanga et al, (1) but with modifications. Briefly, an individual blood spot was punched using a 3mm punch decontaminated first using 10% bleach then followed by 70% ethanol and only with ethanol between all subsequent punches. The spots were placed in individual wells in a 96 well plate. For each plate, four wells set aside for two negative controls (blank filter paper) and two positive controls (DBS prepared from the WHO *Plasmodium falciparum* standard). Each well was then filled with 100 μL of Tris-EDTA (TE) buffer (pH 8.0) and the plate covered with an Eppendorf heat sealing foil (Eppendorf AG, Hamburg Germany) and placed on a shaker for 30 min at a speed of 1000rpm at room temperature. After shaking, the plate was centrifuged at 13000rcf for 5 minutes at 4°C (Eppendorf 5417R). The DNA pellet obtained was washed and re-suspending three times in TE buffer. After the final wash, 10 μL of proteinase K buffer (1.5 mM $MgCl_2$, 50mM KCL, 0.5% Tween 20, 100μg/mL proteinase K and 10mM tris-HCL; pH 8.3) was added to each well and the plate incubated at 55°C for 1 hr followed by a second incubation step at 95°C for 10 minutes to inactivate the proteinase K. The DNA extracts were then stored at -20°C for downstream processing.

*n*PCR-HRM: Extracted DNA was amplified using two sets of primers; PL-1459-F and PL-1706-R for the primary reaction and PL-1473-F and PL-1679-R for the nested amplification reaction. The primary reaction was carried out in a Veriti Thermocycler (Applied Biosystems) with thermal conditions consisting of an initial denaturation step of 95°C for 5 minutes then 35 cycles of denaturation at 94°C for 20 seconds, decreasing annealing temperatures from 65°C to 50°C for 25 seconds (cycles 1-5), 50°C for 40 seconds (cycles 6-10), 50°C for 50 seconds (cycles 11-35), extension at 72°C for 30 seconds, and a final extension at 72°C for 3 minutes. The nested reaction was carried out in a real-time PCR-HRM instrument, Rotor gene (QIAGEN, Germany), with HRM conditions consisting of an incremental temperature increase of 0.2°C from 75°C to 90°C and fluorescence acquisition at each 2 second temperature increment. Representative samples from each cluster of melt curves different from those of the WHO *Plasmodium falciparum* control were obtained cleaned up using the ExoSAP-IT™ protocol and sent for sequencing at Macrogenlab (Seoul, Korea). The sequences obtained were aligned to reference sequences of *Plasmodium ovale* and *malariae* (GenBank: **AB182490** and GenBank: **LT594624**) using Geneious 6.1.6 software.

Chemical analyses

GC/MS Methods for K1: Compounds from a 2µl injection were separated on a SLB-5ms (30m x 0.25mm ID x 0.1µm film thickness; Supelco, USA), using the following temperature program: 35°C for 0.5 min then raised by 7°C/min to 270°C and a constant flow rate of 1.2mL/min of helium. Compounds were detected with an electron impact single quadrapole mass spectrometer (70 eV: ion source 230°C: quadrapole 150°C, mass scan range: 30-350 amu). The analyses were otherwise similar to those described for K2.

Data analyses

Discriminant Analysis of Principal Components (DAPC) was implemented in the adegenet R package v2.0.1 (2-3). The function *find.clusters* was used to determine the optimal number of PCs (to avoid unstable assignments of individuals to clusters, we used a maximum number of PCs corresponding to the sample size divided by three).

For our predictive models, the data was partitioned into training and testing datasets, using 70% and 30% of the data respectively. The training dataset was used to build the model using the methods "Adaboost.M1" (4), "rf" (5) and "rrf" (6) in the R *caret* package (7). Parameters for both models, *ntree* and *mtry* for random forest (rf) and *mfinal* for adaboost were tuned away before running each model, and the best combination of parameters was chosen using the accuracy as the performance metric. This final set of parameters was used to train the final version of the models and their performance were tested on the independent test dataset.

Random forest is a bagging technique based on individual and independent classification trees that are run in parallel with different subsets of the data. In this this ensemble, each tree is built on different bagging subsamples of samples and each split of the tree is constructed with a randomly selected subset of compounds. During the training process, random forest uses the out of bag (OOB) error rate as an estimation of the classification accuracy of the model (5). In Adaboost, on the other hand, the models are trained sequentially and each new model "learns" from the previous one focusing on samples that are difficult to classify (4).

To further explore the date effect in selected compounds, we performed a two-way ANOVA in the R package *ARTool* (8), with infection status (AS + S vs U) or (S vs U) and collection date as main effects (Tables S4-5). In this analysis, all collections on a given date took place at a single location.

Supporting figures and tables:

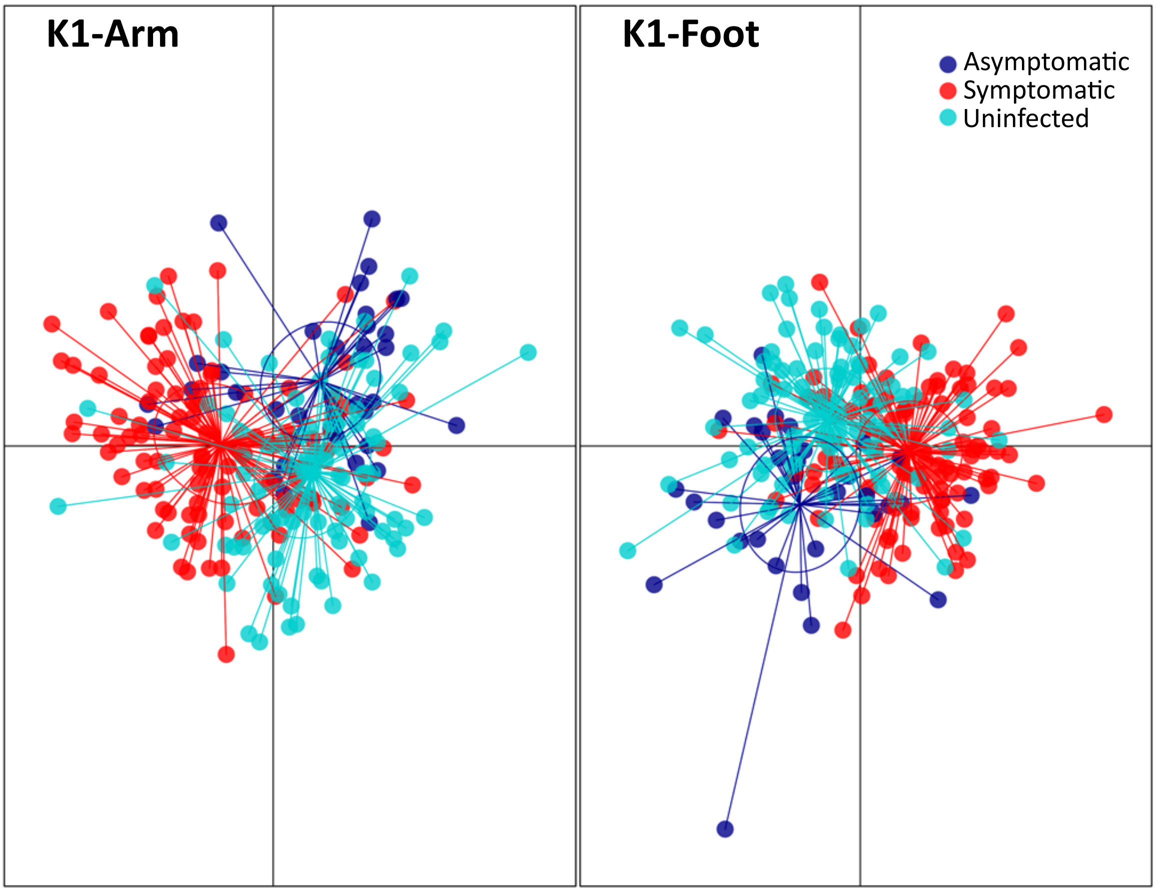

Figure S1. Group separation using DAPC showing differences between Asymptomatic (blue), Symptomatic (red), and Uninfected (cyan) groups for foot and arm volatiles in K1. Vertical line: axis 1 (PC1); horizontal line: axis 2 (PC2). Points represent individual samples, with colours denoting malaria condition and inclusion of 95% inertia ellipses.

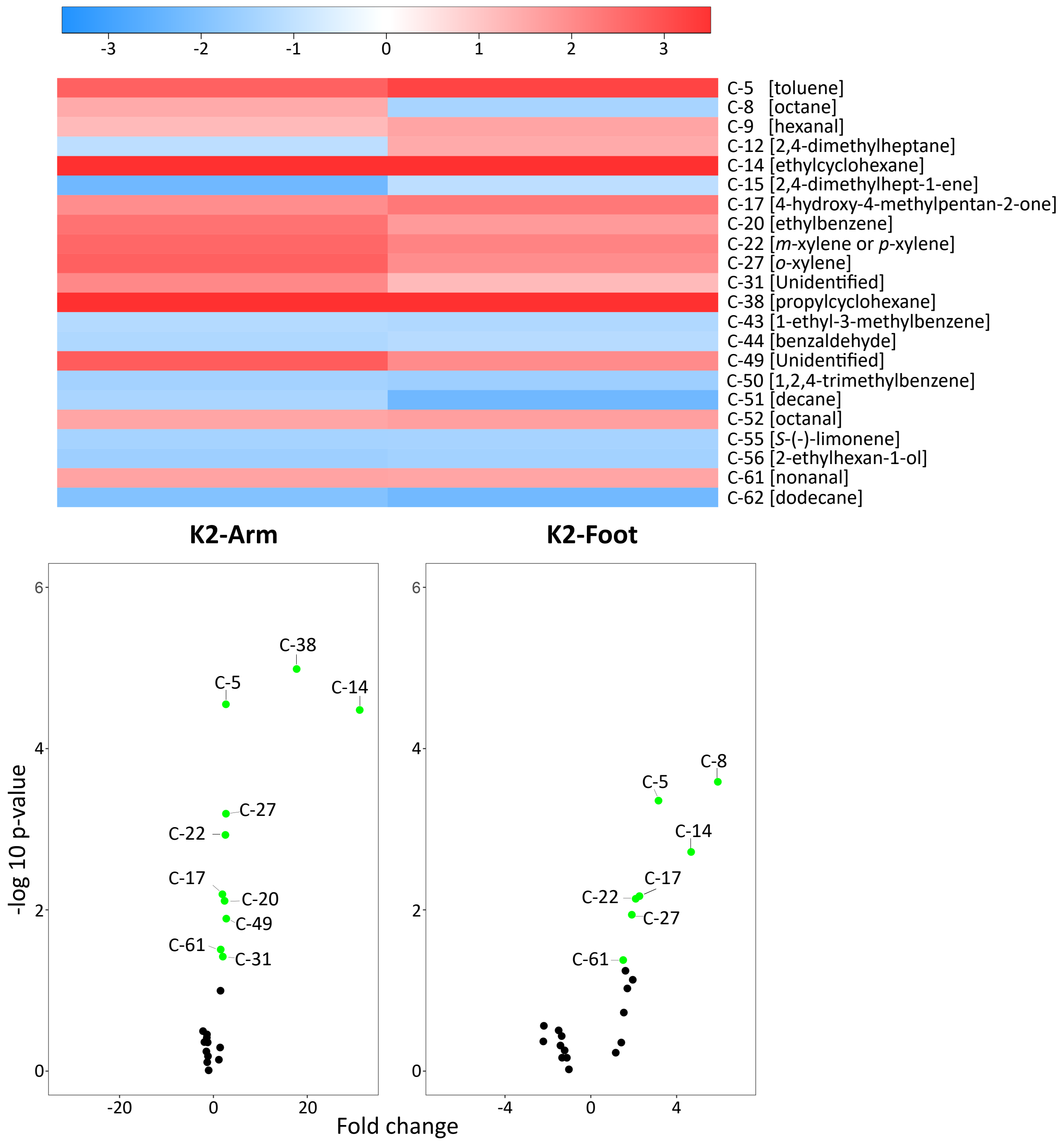

Figure S2. Top: Heatmap showing the fold change of individual compounds in asymptomatic relative to those in symptomatic individuals for K2-arm and K2-foot. Bottom: Volcano plots showing changes in individual compounds in asymptomatic individuals relative to those in symptomatic individuals, with compounds significantly up or down-regulated shown in green (p<0.05 and absolute fold change > 1.5). Non-significant regulated compounds with absolute fold change < 1.5 shown in black.

Table S1. RDT results and sensitivity (% of cases correctly identified by RDT) for symptomatic (S) and asymptomatic (AS) malaria infections, as confirmed by PCR. $AS_{[sub]}$ and $S_{[sub]}$ indicate sub-microscopic infections.

	K1			K2		
	RDT +	RDT -	Sensitivity (%)	RDT +	RDT -	Sensitivity (%)
AS	64	1	98.5	54	0	100
$AS_{[sub]}$	21	15	58.3	19	7	73.1
S	198	1	99.5	67	1	98.5
$S_{[sub]}$	63	24	72.4	82	24	77.4

Table S2. Compounds included in our analyses, with retention time (RT), source and retention index (RI).

No.	Compound	RT	Standard Vendor (Purity)	RI	Published RI
C-5	toluene	6.57	Sigma (99.9%)	762	762
C-8	octane	6.90	Sigma (99%)	797	800
C-9	hexanal	6.93	Sigma (98%)	800	799
C-12	2,4-dimethylheptane	7.25	TCI (98%)	820	822
C-14	ethylcyclohexane	7.49	Sigma (99%)	836	838
C-15	2,4-dimethylhept-1-ene	7.51	TCI (98%)	838	-
C-17	4-hydroxy-4-methylpentan-2-one	7.58	Sigma (99%)	841	840
C-20	ethylbenzene	7.90	Sigma (99%)	862	868
C-22	*m*-xylene or *p*-xylene	8.02	Sigma (99.5%)	870	867 (*m*), 883 (*p*)
C-27	*o*-xylene	8.44	Acros (99%)	897	896
C-31	Unidentified*	8.62		907	
C-38	propylcyclohexane	9.12	Sigma (99%)	932	936
C-43	1-ethyl-3-methylbenzene	9.71	Fluka (99%)	963	968
C-44	benzaldehyde	9.72	Sigma (99%)	963	996
C-49	Unidentified*	10.19		988	
C-50	1,2,4-trimethylbenzene	10.38	Fluka (98.5%)	997	985
C-51	decane	10.45	Sigma (99%)	1003	999
C-52	octanal	10.53	Sigma (99%)	1004	1004
C-55	*S*-(-)-limonene	11.11	Fluka (99%)	1031	1036
C-56	2-ethylhexan-1-ol	11.11	Fluka (99%)	1031	-
C-61	nonanal	12.75	Sigma (95%)	1108	1108
C-62	dodecane	14.70	Fluka (98%)	1201	1199

* indicates compounds could not be acquired commercially
- indicates no published RI available

Table S3 Reported mosquito response to individual compounds

	Compound ID
**C-5**	**toluene**
C-8	octane
**C-9**	**hexanal***[9]
C-12	2,4-dimethylheptane
**C-14**	**ethylcyclohexane**
C-15	2,4-dimethylhept-1-ene
**C-17**	**4-hydroxy-4-methylpentan-2-one**
**C-20**	**ethylbenzene*** [10]
C-22	*m*-xylene or *p*-xylene*[10]
C-27	*o*-xylene
**C-31**	**Unidentified**
**C-38**	**propylcyclohexane**
C-43	1-ethyl-3-methylbenzene
C-44	benzaldehyde*[11-13]
**C-49**	**Unidentified**
C-50	1,2,4-trimethylbenzene*[14]
C-51	decane
C-52	octanal*[11, 14]
C-55	*S*(-)-limonene*[9]
**C-56**	**2-ethylhexan-1-ol*** [11]
**C-61**	**nonanal*** [11]
C-62	dodecane

Asterisks indicate compounds in the literature that elicit electrophysiological or behavioral responses from mosquitoes (with relevant citations provided). Key predictive compounds discussed in the text are listed in bold.

Table S4. Previous reported occurrence and known biological sources of key predictive compounds.

**Compound no.**		**Reported volatile emissions**	**Known biological sources**
C-5	toluene	Human skin (15) and breath (16); *Plasmodium in vitro* (17)	Produced by *Clostridium* and other bacteria genera found in the human gut microbiome (18)
C-9	hexanal	Human skin (15), and *Plasmodium in vitro* (19)	Produced by *Lactobacillus* and *Enterococcus* bacteria (20), which are present in the human gut microbiome (21). Also formed by the peroxidation of fatty acids in cell membrane phospholipids (22)
C-14	ethylcyclohexane	Human breath (23)	Produced by *Lactobacillus* bacteria (24)
C-17	4-hydroxy-4-methylpentan-2-one	Guinea pig (25) and dog (26) skin volatiles; fox odor glands (27)	Cytochrome P450 mediated oxidation of degradation products of squalene (28, 29)
C-20	ethylbenzene	Human breath (30)	Produced by *Streptococcus mutans,* which has been linked to human oral decay (31)
C-31	Unidentified	-	-
C-38	propylcyclohexane	*Actinomycetes* bacteria (32, 33)	Produced by *Actinomycete*s bacteria, which can be present in the human gut microbiome (32, 33)
C-49	Unidentified	-	-
C-56	2-ethylhexan-1-ol	Emitted by *Heliothis virescens* larvae (34) and *Cydia nigricana* moths (35)	Produced by *Streptococcus mutans,* which has been linked to human oral decay (31)
C-61	nonanal	Human skin (15) and breath (23)	Formed by the peroxidation of fatty acids in cell membrane phospholipids (22)

Table S5. Effect of infection status, comparing infected individuals vs uninfected individuals (AS + S vs U) on specific dates, effect of date, and interaction between these factors on emission levels of key predictive compounds. Two-way ANOVA, N=9.

**Compound**	**Malaria status F(1,43) p value**	**Date F(8,43) p value**	**Interaction F(8,43) p value**
C-5 [toluene]	36.87<br>2.90E-07	8.78<br>5.10E-07	15.28<br>2.50E-10
C-9 [hexanal]	25.43<br>8.80E-06	6.17<br>2.70E-05	7.56<br>3.00E-06
C-14 [ethylcylohexane]	36.18<br>3.50E-07	28.70<br>7.60E-15	7.50<br>3.30E-06
C-17 [4-hydroxy-4-methylpentan-2-one]	4.60<br>3.80E-02	12.17<br>6.90E-09	2.01<br>6.80E-02
C-20 [ethylbenzene]	4.69<br>3.60E-02	43.72<br>3.40E-18	5.02<br>2.00E-04
C-31 [Unidentified]	31.86<br>1.20E-06	6.08<br>3.10E-05	6.36<br>2.00E-05
C-38 [propylcyclohexane]	78.26<br>3.10E-11	27.27<br>1.80E-14	8.29<br>1.00E-06
C-49 [Unidentified]	63.41<br>5.30E-10	17.49<br>3.00E-11	7.55<br>3.10E-06
C-56 [2-ethylhexan-1-ol]	14.17<br>5.00E-04	3.01<br>9.00E-03	5.17<br>1.50E-04
C-61 [nonanal]	0.241<br>6.30E-01	2.36<br>3.30E-02	1.43<br>2.10E-01

References

1. Kipanga PN*, et al.* (2014) High-resolution melting analysis reveals low *Plasmodium* parasitaemia infections among microscopically negative febrile patients in western Kenya. *Malar J* 13:429.
2. Jombart T (2008) adegenet: a R package for the multivariate analysis of genetic markers. *Bioinformatics* 24(11):1403-1405.
3. Jombart T & Ahmed I (2011) adegenet 1.3-1: new tools for the analysis of genome-wide SNP data. *Bioinformatics* 27(21):3070-3071.
4. Freund Y & Schapire RE (1996) Experiments with a new boosting algorithm. *Proceedings of the Thirteenth International Conference on Machine Learning* (Morgan Kaufmann, San Fransisco), pp 148-156.
5. Breiman L (2001) Random Forests. *Machine Learning* 45(1):5-32.
6. Houtao D & Runger G (2012) Feature selection via regularized trees (Proceedings of the International Joint Conference on Neural Networks, Brisbane).
7. Kuhn M (2014) Package "caret" version 6.0-79. Available at https://cran.r-project.org/web/packages/caret/caret.pdf.
8. Kay M & Wobbrock J (2016) Package "ARTool" version 0.10 2. Available at https://cran.r-project.org/web/packages/ARTool/ARTool.pdf. doi: 10.5281/zenodo.48543.
9. Nyasembe VO, Teal PE, Mukabana WR, Tumlinson JH, & Torto B (2012) Behavioural response of the malaria vector *Anopheles gambiae* to host plant volatiles and synthetic blends. *Parasit Vectors* 5:234.
10. Ye Z, Liu F, & Liu N (2016) Olfactory responses of southern house mosquito, *Culex quinquefasciatus,* to human odorants. *Chem Senses* 41(5):441-447.
11. Carey AF, Wang G, Su CY, Zwiebel LJ, & Carlson JR (2010) Odorant reception in the malaria mosquito *Anopheles gambiae*. *Nature* 464(7285):66-71.
12. Tauxe GM, MacWilliam D, Boyle SM, Guda T, & Ray A (2013) Targeting a dual detector of skin and CO2 to modify mosquito host seeking. *Cell* 155(6):1365-1379.
13. Odalo JO*, et al.* (2005) Repellency of essential oils of some plants from the Kenyan coast against *Anopheles gambiae*. *Acta Trop* 95(3):210-218.
14. Qiu YT, van Loon JJ, Takken W, Meijerink J, & Smid HM (2006) Olfactory Coding in Antennal Neurons of the Malaria Mosquito, *Anopheles gambiae*. *Chem Senses* 31(9):845-863.
15. Jiang R, Cudjoe E, Bojko B, Abaffy T, & Pawliszyn J (2013) A non-invasive method for in vivo skin volatile compounds sampling. *Anal Chim Acta* 804:111-119.
16. Peng G*, et al.* (2010) Detection of lung, breast, colorectal, and prostate cancers from exhaled breath using a single array of nanosensors. *Br J Cancer* 103(4):542-551.
17. Kelly M*, et al.* (2015) Malaria parasites produce volatile mosquito attractants. *mBio* 6(2).
18. Zargar K*, et al.* (2016) In vitro characterization of phenylacetate decarboxylase, a novel enzyme catalyzing toluene biosynthesis in an anaerobic microbial community. *Sci Rep* 6:31362.
19. Correa R, Coronado LM, Garrido AC, Durant-Archibold AA, & Spadafora C (2017) Volatile organic compounds associated with *Plasmodium falciparum* infection *in vitro*. *Parasit Vectors* 10:215.
20. Randazzo CL, Pitino I, De Luca S, Scifò GO, & Caggia C (2008) Effect of wild strains used as starter cultures and adjunct cultures on the volatile compounds of the Pecorino Siciliano cheese. *Int J Food Microbiol* 122(3):269-278.
21. Yatsunenko T*, et al.* (2012) Human gut microbiome viewed across age and geography. *Nature* 486(7402):222-227.

22. Poli D*, et al.* (2010) Determination of aldehydes in exhaled breath of patients with lung cancer by means of on-fiber-derivatisation SPME–GC/MS. *J Chromatogr B Analyt Technol Biomed Life Sci* 878(27):2643-2651.
23. Costello BdL*, et al.* (2014) A review of the volatiles from the healthy human body. *J Breath Res* 8(1):014001.
24. Di Cagno R*, et al.* (2011) Manufacture of Italian Caciotta-type cheeses with adjuncts and attenuated adjuncts of selected non-starter lactobacilli. *Int Dairy J* 21(4):254-260.
25. Omrani SM*, et al.* (2010) Differential responses of *Anopheles stephensi* (Diptera: Culicidae) to skin emanations of a man, a cow, and a guinea pig in the olfactometer. *Iranian Journal of Arthropod-borne Diseases* 4(1):1-16.
26. Holderman CJ, Kaufman PE, Booth MM, & Bernier UR (2017) Novel collection method for volatile organic compounds (VOCs) from dogs. *J Chromatogr B Analyt Technol Biomed Life Sci* 1061-1062:1-4.
27. Lorenzo MG, Vidal DM, & Zarbin PHG (2014) Control of neglected disease insect vectors: future prospects for the use of tools based on behavior manipulation-interference. *Journal of the Brazilian Chemical Society* 25:1799-1809.
28. Cooper HLR & Groves JT (2011) Molecular probes of the mechanism of cytochrome P450. Oxygen traps a substrate radical intermediate. *Arch Biochem Biophys* 507(1):111-118.
29. Stein RA & Mead JF (1988) Small hydrocarbons formed by the peroxidation of squalene. *Chem Phys Lipids* 46(2):117-120.
30. Fenske JD & Paulson SE (1999) Human breath emissions of VOCs. *J Air Waste Manag Assoc* 49(5):594-598.
31. Hertel M*, et al.* (2016) Detection of signature volatiles for cariogenic microorganisms. *Eur J Clin Microbiol Infect Dis* 35(2):235-244.
32. Citron CA, Barra L, Wink J, & Dickschat JS (2015) Volatiles from nineteen recently genome sequenced actinomycetes. *Org Biomol Chem* 13(9):2673-2683.
33. Citron CA, Rabe P, & Dickschat JS (2012) The scent of bacteria: headspace analysis for the discovery of natural products. *Journal of Natural Products* 75(10):1765-1776.
34. Morawo T & Fadamiro H (2016) Identification of key plant-associated volatiles emitted by *Heliothis virescens* larvae that attract the parasitoid, *Microplitis croceipes*: implications for parasitoid perception of odor blends. *J Chem Ecol* 42(11):1112-1121.
35. Dalen M, Knudsen GK, Norli HR, & Thöming G (2015) Sources of volatiles mediating host location behaviour of *Glypta haesitator*, a larval parasitoid of *Cydia nigricana*. *Biological Control* 90:128-140.
